# Interactions count: plant origin, herbivory and disturbance jointly explain seedling recruitment and community structure

**DOI:** 10.1038/s41598-017-08401-3

**Published:** 2017-08-15

**Authors:** Lotte Korell, Birgit R. Lang, Isabell Hensen, Harald Auge, Helge Bruelheide

**Affiliations:** 1 Institute of Biology, Martin Luther University Halle-Wittenberg, Am Kirchtor 1, D-06108 Halle, Germany; 2 Department of Community Ecology, Helmholtz Centre for Environmental Research (UFZ), Theodor-Lieser-Straße 4, D-06120 Halle, Germany; 3 German Centre for Integrative Biodiversity Research (iDiv) Halle-Jena-Leipzig, Deutscher Platz 5e, D-4103 Leipzig, Germany; 4 Institute of Special Botany, Philosophenweg 16, D-07743 Jena, Germany

## Abstract

Herbivory and disturbance are major drivers of biological invasions, but it is unclear how they interact to determine exotic vs. native seedling recruitment and what consequences arise for biodiversity and ecosystem functioning. Previous studies neglected the roles of different, potentially interacting, guilds of generalist herbivores such as rodents and gastropods. We therefore set up a full-factorial rodent exclusion x gastropod exclusion x disturbance x seed-addition experiment in a grassland community in Central Germany and measured early seedling recruitment, as well as species richness, species composition and aboveground biomass. Gastropod herbivory reduced the positive effect of disturbance on seedling recruitment, particularly for exotic species. Rodent herbivory had weak positive effects on seedling recruitment at undisturbed sites, irrespective of species origin. This effect was likely driven by their strong negative effect on productivity. Interactive effects between both herbivore guilds became only evident for species richness and composition. How many species established themselves depended on disturbance, but was independent of species origin. The fewer exotic species that established themselves increased productivity to a stronger extent compared to native species. Our study highlights that joint effects of disturbance, herbivory and species origin shape early recruitment, while they only weakly affect biodiversity and ecosystem functioning.

## Introduction

Biological invasions are a major component of global change and known to alter biodiversity and ecosystem functions of plant communities^1^. Despite increasing evidence that biological invasions are driven by the combination of different processes, most studies so far have focused mainly on one or a few processes and rarely, if ever, consider their interactions^2^.

To initially colonize a given site and to become established, seedling recruitment is of high importance for exotic as well as for native species^3,4^. Herbivory and disturbance shape seedling recruitment in plant communities^5,6^, and thus, their roles in driving invasion success or failure have been extensively studied and discussed^7–9^. The enemy release hypothesis^10^ suggests that exotic species gain advantage over native species because they are released from their co-evolved specialist herbivores, and are less attacked by resident generalist herbivores compared to native species, e.g. because they produce novel defenses^11^. In contrast, the biotic resistance hypothesis^12^ states that resident generalist herbivores may constrain exotic plant invasions by suppressing those exotic species that have not evolved effective defense mechanisms against them. However, so far studies on the effects of generalist herbivores on early seedling recruitment and establishment success have provided inconsistent results, as they found stronger^13,14^, equal^2,15,16^ or weaker^17,18^ effects on exotic compared to native species. This apparent inconsistency may at least be partly explained by the different feeding preferences of the key herbivores in the particular study system.

Two important generalist herbivore guilds of Central European grasslands, that may show such specific feeding preferences, are gastropods and rodents. Feeding preferences of generalists depend on various plant traits, such as carbon/nitrogen ratio, specific leaf area, leaf toughness and chemical defenses^19^, which, however, do not only differ between exotic and native plant species but also between functional groups and across life-cycle stages of plants^20,21^. A consequence of such feeding preferences may be non-additive effects of herbivore guilds, e.g. on early establishment^22^ or community structure^23^. Gastropods often prefer seedlings over adult plants and legumes or non-legume herbs over grasses^16,24^. Feeding preferences of rodents differ between granivorous and herbivorous rodents: while granivorous rodents are known to preferentially consume seeds of large-seeded species, herbivorous rodents such as voles consume biomass of grasses that is easily available^25^, or of legumes that is highly nutritious^26^.

There are comparably few studies on the importance of generalist herbivores for biological invasions, and those mostly concentrated on granivorous rodents in North America^13,14^. In contrast to North American grasslands, where granivores were found to play a key role in structuring plant communities^27^, many studies in Central European grasslands pointed out the importance of gastropods and herbivorous rodents^16,23,28^. Granivorous rodents can mediate biotic resistance against exotic plant invasions by particularly suppressing early recruitment or population growth of large-seeded weak invaders^13^. In contrast, in a previous study^16^, herbivorous rodents were found to equally affect seedling recruitment of exotic and native plant species. The very few studies on the effect of gastropod herbivory showed that they either equally suppressed native and exotic species^15,16^ or preferred native over exotic plant species^17,29^. Thus, there is evidence that the outcome of biological invasions differs, depending on the groups of the dominant herbivores in the system.

Another factor suggested to drive invasion success of exotic plant species is disturbance which removes resident competitors^9,30^. Disturbance of the vegetation cover and exposure of soil (in the following disturbance), for example through human activities such as ploughing, increases the availability of microsites and thereby provides invasion opportunities for exotic species^31,32^. Compared to native species exotic species are suggested to have a superior ability to exploit resources released by disturbance^30^. It was, however, also found that native species similarly benefitted from disturbance as exotic species^5,6^. Therefore, controlled experiments are needed where exotic and native species are introduced to sites with both intact and disturbed vegetation. The few studies using this approach, though, provided inconsistent results, as exotic species were found to have lower^20^, equal^2^ or higher seedling recruitment compared to native species^18^.

Disturbance and herbivory may not act independently, but interactively determine establishment success of plant species: if competitors are removed by disturbance and germination is no longer microsite-limited, the effects of herbivory on seedling recruitment should be greater compared to undisturbed sites^33^. Despite the knowledge of potentially interactive effects of disturbance and herbivory, it is still unclear how both processes jointly affect the establishment success of exotic compared to native species. Indeed, a study by Maron, *et al*.^18^ showed that the joint effects of disturbance and rodent granivory on seedling recruitment varies with species origin, but no such three-way interaction was found by Mueller, *et al*.^2^ for invertebrate herbivory. Again, these results may depend on the key herbivores in the study system (i.e. herbivorous rodents, granivorous rodents, gastropods), and thus, it is important to disentangle effects of different herbivore guilds and disturbance on exotic vs. native plant species.

Studies on the role of disturbance and herbivory for biological invasions either focused on early seedling recruitment or on community structure and ecosystem functioning^32,34,35^. The seedling stage is a vulnerable time in the life-cycle of plants^36^ and changes in seedling recruitment as an effect of disturbance or herbivory can influence plant abundance, community composition, species richness and productivity (reviewed in refs^6,33^). However, it is not known whether complex interactions that are evident during early seedling recruitment result in changes in species richness, community composition and productivity. Maron, *et al*.^27^ showed that the positive impact of exotic vs. native species on the productivity of the plant community was enhanced when local filters such as competition from residents and generalist herbivory were removed. Moreover, their finding that exotic species increased productivity to larger amounts compared to native species in spite of similar species richness^27^, suggests that different mechanisms underlie the diversity-productivity relationship in exotic and native dominated communities^37–39^. While it is generally assumed that exotic species directly contribute to productivity through intrinsically higher growth rates which are related to faster resource uptake and use^40^, the diversity-productivity relationship may also be shaped by selective herbivory on exotic legumes and herbs^41^.

To address these multiple interactions we devised a full-factorial experiment in a grassland community in Central Germany, where we excluded rodents and gastropods, and added seeds of 20 exotic and native species to plots that were either subjected to a soil disturbance treatment or remained undisturbed. Specifically we seek to answer the following research questions:

1. *How do disturbance and the different herbivore guilds, separately and interactively, affect the establishment of exotic vs. native plant species?* We expect exotic species, compared to native species, to benefit more from disturbance and to be less affected by gastropods and equally affected by herbivorous rodents. Moreover, we expect that disturbance and herbivory interact with each other and in combination change the establishment of exotic and native species.
2. *What are the consequences for species diversity, composition and productivity of plant communities that are experimentally invaded by exotic and native species?* We expect that changes in seedling recruitment of exotic vs. native species as an effect of disturbance, gastropod and rodent herbivory result in changes in diversity, composition and productivity over time. Moreover, we expect that, compared to native species, introductions of exotic species lead to a stronger increase in productivity, while species richness generally increases, irrespective of species origin.

## Results

### How do disturbance and the different herbivore guilds, separately and interactively, affect the establishment of exotic vs. native plant species?

Out of the 40 species sown 33 species (14 exotic, 19 native) germinated and more than 4,000 seedlings recruited from sown seeds. Disturbance substantially enhanced seedling recruitment, both in terms of the number of species with seedling recruitment (disturbed: 10.4 ± 0.7, undisturbed: 6.8 ± 0.7; mean ± SE) and the proportion of seedling recruitment (disturbed: 6.4 ± 1.2%, undisturbed: 2.4 ± 1.2%). However, for the proportion of seedling recruitment this effect differed between functional groups and species origins (Table 1). Specifically, disturbance tended to decrease seedling recruitment for exotic legumes (exotic legumes disturbed: 5.2 ± 2.9%, exotic legumes undisturbed: 6.9 ± 2.9%) while native legumes were not affected by disturbance (native legumes disturbed: 0.4 ± 2.2%, native legumes undisturbed: 0.5 ± 2.2%). Moreover, a strong positive effect of disturbance on the proportion of seedling recruitment was much more pronounced for exotic grasses (exotic grasses disturbed: 18.7 ± 2.9%, exotic grasses undisturbed: 2.5 ± 2.9%) and herbs (exotic herbs disturbed: 5.2 ± 1.4%, exotic herbs undisturbed: 2.5 ± 1.4%) compared to native grasses (native grasses disturbed: 8.9 ± 2.9%, native grasses undisturbed: 1.8 ± 2.9%) and herbs (native herbs disturbed: 2.4 ± 1.3%, native herbs undisturbed: 0.9 ± 1.3%; Table 1; marginally significant interaction disturbance x functional group x species origin).

**Table 1:** Results of linear mixed models for the number of species with seedling recruitment and for the proportion of seedling recruitment (i.e the numbers of recruited individuals in relation to the number of added propagules per species) testing for the effects of rodent herbivory (rodent control, rodent exclusion), gastropod herbivory (gastropod control, gastropod exclusion), species origin (exotic = E, native = N), and disturbance (disturbed =  + D, undisturbed = −D).

Fixed effects	Number of species with seedling recruitment		Proportion of seedling recruitment	
	*D.f*.	*F* ratio	*D.f*.	*F* ratio
Rodent (ROD)	1, 4	1.58	1, 4	0.82
Gastropod (GAS)	1, 8	0.01	1, 8	7.07*
Species origin (SO)	1, 48	0.16	1, 28	2.11
Disturbance (DIS)	1, 48	50.73***	1, 28	50.9***
Functional group (FG)	—	—	2, 28	**2.81+**
ROD × GAS	1, 8	1.18	1, 8	0.51
ROD × SO	1, 48	0.04	1, 28	0.08
ROD × DIS	1, 48	4.74*	1, 28	1.96
ROD × FG	—	—	2, 28	**2.51+**
GAS × SO	1, 48	1.18	1, 28	5.46*
GAS|E	—	—	1, 28	12.08**
GAS|N	—	—	1,28	0.05
GAS × DIS	1, 48	1.65	1, 28	**3.91+**
GAS × FG	—	—	2, 28	7.54**
GAS|Gr	—	—	1, 28	0.38
GAS|Lg	—	—	1, 28	0.18
GAS|Hb	—	—	1, 28	**3.00+**
SO × DIS	1, 48	0.98	1, 28	0.14
SO × FG	—	—	2, 28	0.79
DIS × FG	—	—	2, 28	31.1***
ROD × GAS × SO	1, 48	0.09	1, 28	1.29
ROD × GAS × DIS	1, 48	1.18	1, 28	1.77
ROD × GAS × FG	—	—	2, 28	0.4
ROD × SO × DIS	1, 48	0	1, 28	0.82
ROD × SO × FG	—	—	2, 28	0.12
ROD × DIS × FG	—	—	2, 28	1.35
GAS × SO × DIS	1, 48	2.83	1, 28	5.85*
GAS||E, +D	—	—	1, 28	21.28***
GAS|N, +D	—	—	1, 28	0
GAS|E, −D	—	—	1, 28	0.09
GAS|N, −D	—	—	1, 28	0.15
GAS × SO × FG	—	—	2, 28	4.18*
GAS|E, Gr	—	—	1, 28	0.67
GAS|N, Gr	—	—	1, 28	0.62
GAS|E, Lg	—	—	1, 28	24.05***
GAS|N, Lg	—	—	1, 28	0.14
GAS|E, Hb	—	—	1, 28	0.63
GAS|N, Hb	—	—	1, 28	0.14
GAS × DIS × FG	—	—	2, 28	3.73*
GAS|Gr, +D	—	—	1, 28	0.41
GAS|Gr, −D	—	—	1, 28	0.42
GAS|Lg, +D	—	—	1, 28	25.11***
GAS|Lg, −D	—	—	1, 28	0.32
GAS|Hb, +D	—	—	1, 28	0.49
GAS|Hb, −D	—	—	1, 28	0.42
SO × DIS × FG	—	—	2, 28	**2.8+**
DIS|E, Gr	—	—	1, 28	56.85***
DIS|N, Gr	—	—	1, 28	25.61***
DIS|E, Lg	—	—	1, 28	**3.64+**
DIS|N, Lg	—	—	1, 28	0
DIS|E, Hb	—	—	1, 28	30.08***
DIS|N, Hb	—	—	1, 28	26.33***
ROD × GAS × SO × DIS	1, 48	0.48	1, 28	1.43
ROD × GAS × SO × FG	—	—	2, 28	0.83

Functional group (grasses = Gr, legumes = Lg, herbs = Hb) and its interactions were not included in the model for the number of species with seedling recruitment. For relevant interactions, the effect of one factors is tested for each level of the other factor or factorial combinations (simple main effects). *D.f*. refers to the numerator and denominator degrees of freedom. Significant F values are shown in bold: ^+^*P* < 0.10, *P < 0.05, **P < 0.01, ***P < 0.001.

**Figure 1:**
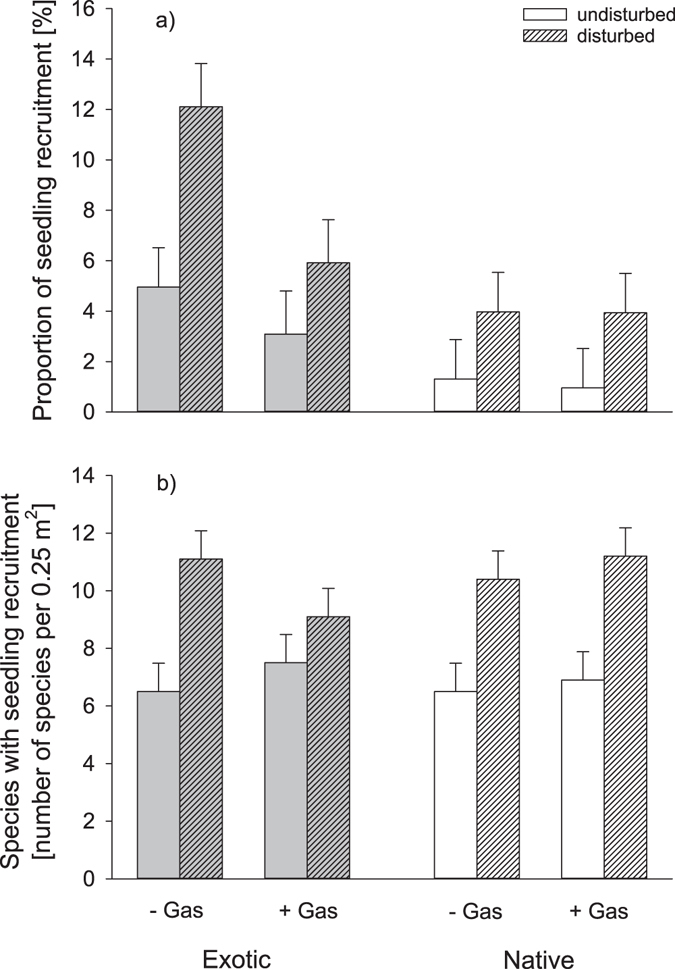
Effect of gastropod herbivory (+Gas = gastropod control, −Gas = gastropod exclusion), disturbance (disturbed, undisturbed) and species origin (exotic, native) on (**a**) the proportion of seedling recruitment (number of recruited seedlings divided by the number of added seeds) and (**b**) the number of species with seedling recruitment. Note that data shown are the untransformed least square means ± SE of a linear mixed model.

**Figure 2:**
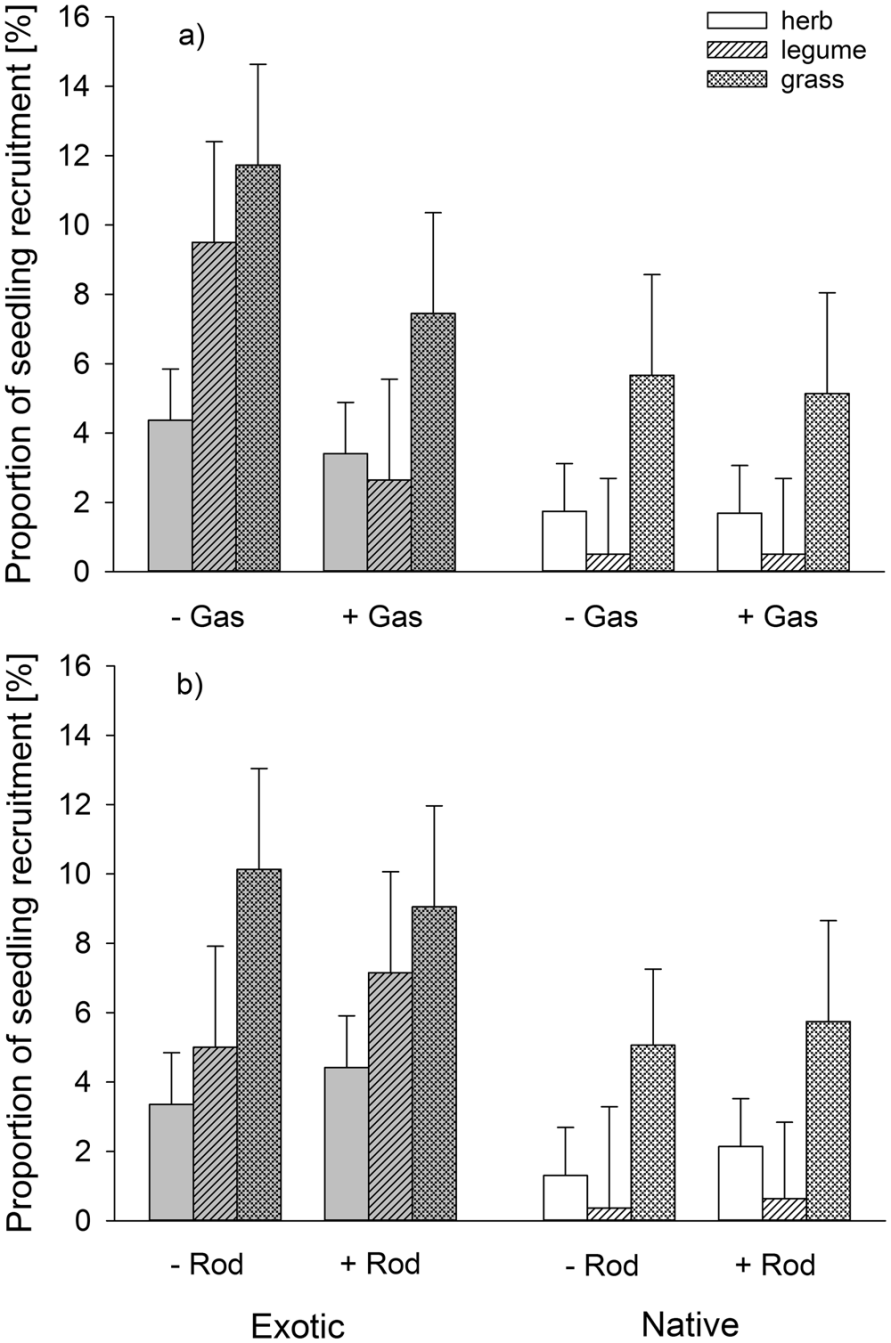
(**a**) Effect of gastropod herbivory (+Gas = gastropod control, −Gas = gastropod exclusion), disturbance (disturbed, undisturbed) and species origin (exotic, native) on the on the proportion of seedling recruitment (number of recruited seedlings divided by the number of added seeds), (**b**) effect of rodent herbivory (+Rod = rodent control, −Rod = rodent exclusion) disturbance (disturbed, undisturbed) and species origin (exotic, native) on the proportion of seedling recruitment (number of recruited seedlings divided by the number of added seeds). Note that data shown are the untransformed least square means ± SE of a linear mixed model.

Gastropod herbivory reduced the proportion of seedling recruitment (gastropod exclusion: 3.4 ± 1.2%, gastropod control: 5.5 ± 1.2%) but not the number of species with seedling recruitment (Table 1). Seedling recruitment of legumes was significantly reduced by gastropod herbivory in disturbed (legumes disturbed gastropod exclusion: 5.5 ± 2.0%, legumes disturbed gastropod control: 0.5 ± 2.0%) compared to undisturbed plots (legumes undisturbed gastropod exclusion: 4.8 ± 2.0%, legumes undisturbed gastropod control: 2.6 ± 2.0%; Table 1, significant interaction gastropod x disturbance x functional group). Moreover, gastropod herbivory reduced seedling recruitment of exotic species (exotics gastropod exclusion: 8.5 ± 1.6%, exotics gastropod control: 4.5 ± 1.6%), to a stronger extent as compared to native seedling densities (natives gastropod exclusion: 2.4 ± 1.4%, natives gastropod control: 2.6 ± 1.4%; Table 1, significant interaction gastropod x species origin). The interaction between gastropod herbivory and species origin, however, also varied between disturbed and undisturbed plots (Fig. 1a), as well as between the three functional groups (Fig. 2a). Gastropod herbivory reduced the positive effect of disturbance on seedling densities only for exotic but not for native species (Table 1, significant interaction gastropods x disturbance x species origin; Fig. 1a). The effects of disturbance and gastropod herbivory on the number of exotic and native species with seedling recruitment showed the same direction, but were not significant (Table 1, Fig. 1b). Nevertheless, compared to native legumes, the number of exotic legumes was lower in plots with gastropod herbivory while neither recruitment of exotic nor native grasses or non-legume herbs showed significant responses to gastropod herbivory (Table 1, significant three-way interaction gastropod x species origin x functional group; Fig. 2a).

Overall, rodent herbivory had only weak effects on seedling recruitment. In contrast to expectations, the presence and not the absence of rodent herbivory increased the number of species with seedling recruitment by about two species at undisturbed (rodent exclusion undisturbed: 5.8 ± 0.8, rodent control undisturbed: 7.9 ± 0.8) compared to disturbed plots (rodent exclusion disturbed: 10.5 ± 0.8, rodent control disturbed: 10.4 ± 0.8, Table 1). Moreover, irrespective of species origin, the presence of rodents marginally increased the proportion of seedling recruitment of non-legume herbs, while there were was no such effect on grasses and legumes (Table 1, significant interaction of rodent herbivory x functional group; Fig. 2b).

### What are the consequences for species diversity, composition and productivity of plant communities experimentally invaded by exotic and native species?

**Figure 3:**
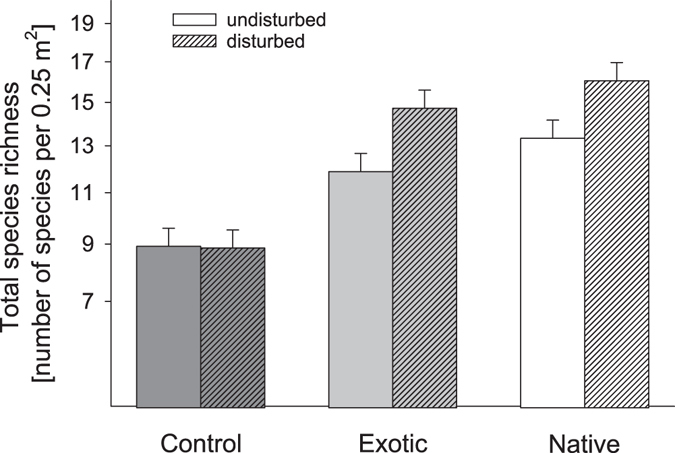
Effect of seed addition (control = no seed addition, exotic = exotic seed addition, native = native seed addition) and disturbance (undisturbed, disturbed) on total species richness (including resident and sown species). Data shown are least square means of repeated-measure linear mixed models ± SE averaged across two years. Note that species richness is given on a square root scale.

Generally, with seed addition total species richness was increased by about five species and with disturbance by about one species per 0.25 m^2^ plot (Table 2). Regardless of species origin disturbance in combination with seed addition increased total species richness by about three species (Fig. 3), and herb species richness about five species (Table 2). Across both study years (2012 and 2013) disturbance substantially changed the composition of the plant community (Fig. S1 and Table S1). The indicator species analysis showed that, consistently across both years, the exotic herb *Dianthus giganteus* benefitted the most from disturbance (Table S2). Furthermore, the grasses *Bromus inermis* and *Bromus hordeaceus* were indicators of disturbance in the first year, while the herbs *Dipsacus sylvestris* and *Sanguisorba minor* were associated with disturbance in the second year. However, disturbance and seed addition of exotic and native species also interacted with each other and community composition was shifted towards sown exotic and native species particularly at disturbed plots (Fig. S1 and Table S1). Disturbance substantially reduced aboveground productivity by 30% (Table 2).

**Table 2:** Results of linear mixed models testing for the effect of rodent herbivory (rodent control, rodent exclusion), gastropod herbivory (gastropod control, gastropod exclusion), species origin (no seed addition control = C, exotic seed addition = E, native seed addition = N), and disturbance (disturbed = +D, undisturbed = −D) on total, grass, herb and legume species richness and aboveground productivity.

Fixed effects	*D.f*.	Species richness				Abovegr. productivity
		Total	Grasses	Herbs	Legumes	
		*F* ratio	*F* ratio	*F* ratio	*F* ratio	*F* ratio
Rodent (ROD)	1, 4	0.52	2.82	0.92	0.34	10.91*
Gastropod (GAS)	1, 8	**7.67**	0.34	1.88	9.84*	7.93*
Species origin (SO)	2, 80	83.62***	14.29***	64.90***	16.21***	10.25***
‘C vs. E + N’	1,80	159.76***	27.86***	119.24***	31.97***	16.81***
‘E vs. N’	1,80	7.49**	0.72	10.55**	0.46	**3.69+**
Disturbance (DIS)	1, 80	43.27***	0.71	51.63***	11.82***	58.75***
ROD × GAS	1, 8	**5.22+**	6.44*	1.98	0.03	0.75
ROD × SO	2, 80	1.68	0.59	1.64	1.15	2.35
ROD × DIS	1, 80	0	0.03	0.23	0.2	0.41
GAS × SO	2, 80	0.67	0.12	0.52	0	0.61
GAS × DIS	1, 80	**3.38+**	0.48	**3.1+**	**3.27+**	0.18
SO × DIS	2, 80	5.20**	2.06	4.08*	1.49	1.08
‘+D vs. –D’ × ‘C vs. E + N’	1,80	10.33**	—	8.15*	—	—
‘+D vs. +D’ × ‘E vs. N’	1,80	0.08	—	0.02	—	—
ROD × GAS × SO	2, 80	0.55	1.49	0.13	0.47	0.24
ROD × GAS × DIS	1, 80	0.16	1.65	0.02	6.25*	0.61
ROD × SO × DIS	2, 80	0.51	0.54	0.33	0.81	0.37
GAS × SO × DIS	2, 80	0.36	0.12	0.37	0.2	0.27
ROD × GAS × SO × DIS	2, 80	2.03	**2.89+**	1.02	0.45	0.5

Relevant effects are decomposed into orthogonal contrasts to investigate more detailed a-priori hypotheses. Repeated measurements of the same sub-subplots are considered as random effects in all models. *D.f*. gives the numerator and denominator degrees of freedom. ‘Significant’ F values are shown in bold: ^+^*P* < 0.10, *P < 0.05, **P < 0.01, ***P < 0.001.

Gastropod herbivory significantly decreased total species richness by about one species and tended to reduce the positive effect of disturbance on total, legume and herb species richness (marginally significant interaction gastropod herbivory x disturbance, Table 2). Gastropod herbivory affected community composition in the second study year only, characterized by a decrease in the abundance of the resident legume species *Trifolium pratense*. Gastropod herbivory led to a moderate decrease in productivity by about 12%. In contrast to seedling recruitment the effect of gastropod herbivory on species richness and productivity was independent of species origin. Rodent herbivory alone had no effects on species richness, but community composition was affected by rodent herbivory across both study years (Table S1). This finding was also supported by the indicator species analysis (Table S2). *Trifolium dubium* was associated with rodent exclusion in the first study year, while the resident species *Achillea millefolium* and the sown native species *Lactuca serriola* were associated with the presence of rodents in the first and second study year, respectively.

**Figure 4:**
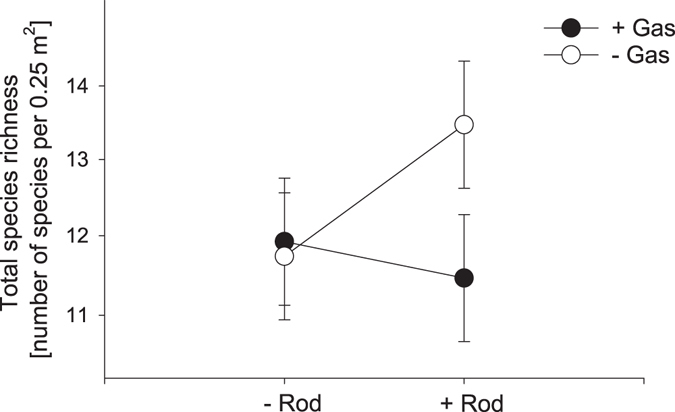
Effect of gastropod herbivory (+Gas = gastropod control, −Gas = gastropod exclusion) and rodent herbivory (+Rod = rodent control, −Rod = rodent exclosure) on total species richness (including resident and sown species). Data shown are least square means of repeated-measure linear mixed models ± SE averaged across two years. Note that species richness is given on a square root scale.

Rodent and gastropod herbivory interacted to determine the species richness of our grassland community: rodent herbivory enhanced total species richness by about two species only in the absence of gastropod herbivory (Fig. 4). Moreover, rodent herbivory decreased the number of grasses in the presence of gastropod herbivory, although this effect was small (rodent control gastropod exclusion: 3.6 ± 1.1, rodent control gastropod control: 2.9 ± 1.1). These interactions between both herbivore guilds were also reflected in slight changes of the community composition within the first study year (Table S1), but there was no significant indicator species for this interaction.

For the species richness of legumes the direction of the rodent x gastropod herbivory interaction was additionally dependent on disturbance (Table 2): at disturbed plots, both gastropods and rodents reduced the number of legume species and their combined effects were mainly additive, while at undisturbed plots particularly the presence of rodents in the absence of gastropods increased the number of legume species compared to the absence of either rodents alone or both herbivore guilds.

**Figure 5:**
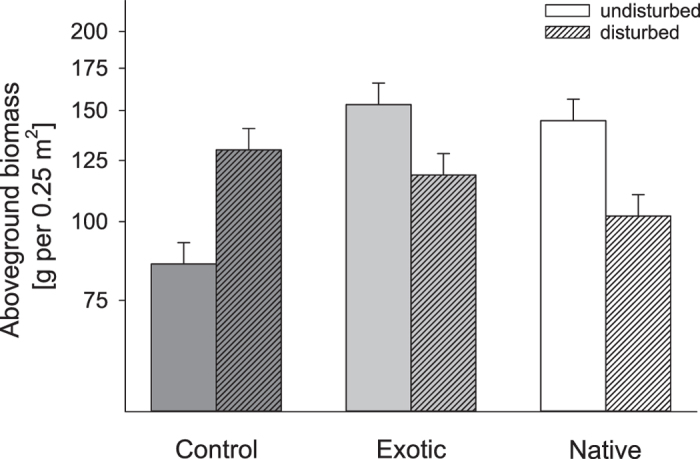
Effect of species origin (control = no seed addition, exotic = exotic seed addition, native = native seed addition) and disturbance (undisturbed, disturbed) on aboveground biomass. Data shown are least square means of repeated-measure linear mixed models ± SE averaged across two years. Note that aboveground biomass is given on a logarithmic scale.

Aboveground productivity was generally increased by seed addition, with exotic seed addition plots displaying a marginally significant higher increase in productivity than native seed addition plots (Fig. 5). Seed addition of native species increased total species richness more strongly than exotic seed addition (natives: 14.6 ± 0.7, exotics: 13.1 ± 0.7). When separating the total species richness into species richness of the three functional groups, particularly the number of native compared to exotic herbs (natives: 8.2 ± 0.5, exotics: 6.9 ± 0.4) increased following seed addition but not grasses or legumes. Moreover, plots experimentally invaded by exotic and native species changed in community composition (Fig. S1 and Table S1).

## Discussion

Our experiment provided important insights into processes determining seedling recruitment of native and exotic species as well as into the consequences for species richness, species composition and productivity. Against expectations, gastropod herbivory reduced the positive effect of disturbance during seedling recruitment for exotic but not for native species. Independent of species origin, rodent herbivory had weak but positive effects on seedling recruitment, particularly on that of herbs and on undisturbed plots. Contrary to the effects observed for seedling recruitment, interactive effects between gastropod herbivory, disturbance and species origin were not evident for species richness, composition and productivity. However, we found interactive effects of gastropod and rodent herbivory on community composition and species richness, which were not previously found on seedling recruitment. As expected, addition of exotic species led to a stronger increase in aboveground productivity compared to native seed addition, while the opposite was true for species richness, suggesting that the relationship between productivity and species richness was different for native and exotic species.

### How do disturbance and the different herbivore guilds, separately and interactively, affect the establishment of exotic vs. native plant species?

As expected, the positive effect of disturbance on seedling recruitment was stronger for exotic compared to native species, which is in line with a study by Maron, *et al*.^18^ (but see refs^2,20^). However, this effect also depended on the affiliation to functional groups, as only exotic herbs and grasses responded positive to soil disturbance, while exotic legumes responded negative to soil disturbance. We can only speculate here, but a higher resource use efficiency and colonization ability of exotic species^30,40^ together with the different resource use strategies of the different functional groups (i.e. nitrogen-fixing legumes vs. non-fixing herbs and grasses) might explain the pattern observed in this study. However, we also have to note that the effect of disturbance on exotic vs. native species affiliated to different functional groups was not independent of gastropod herbivory.

Against our expectations and results from previous studies, gastropods suppressed exotic over native seedling recruitment. Thus, our study is the first one to demonstrate that selective feeding of gastropods on exotic species may represent a strong form of biotic resistance, at least during the establishment phase of exotic plants^9,12^. Cates and Orians^42^ found that exotic, early successional species were preferred by slugs in palatability tests, but whether such preferences result in different seedling densities in the field has only rarely been investigated^2,15,16^. Biotic resistance by gastropods against exotic plant species was mainly driven by the strong herbivore pressure on exotic legumes, which, in the case of the species included in our experiment, were all intentionally introduced as forage or cover crops^43^. We may therefore assume a human pre-selection of these species for high productivity and faster growth^44^, being associated with lower levels of resistance but probably higher levels of tolerance^45^. Lower herbivore resistance would be in line with the preference of gastropods for these exotics. Another important finding of our study was that gastropods reduced seedling recruitment of exotic legumes particularly at disturbed plots, which also explains the negative response of exotic legumes to disturbance (see above). Thus, gastropods can even diminish invasion opportunities provided by disturbances of the vegetation cover^30,31^. A possible mechanism underlying this result may be that disturbance increased the apparency of palatable exotic species for gastropods^46^.

In contrast to the strong negative effects of gastropod herbivory on seedling recruitment of exotic species, rodent herbivory had weak but positive effects on seedling densities of herbs, irrespective of their origin. We have to mention here, that due to the split-plot design of our study we had fewer replicates for the factor rodent herbivory (5 replicates) as it represents the main-plot level, compared to the factor gastropod herbivory (10 replicates) as it represents the subplot level, which leads to a lower power to detect significant effects of rodents compared to gastropods. Moreover, the presence of rodents increased the opportunities for sown species, in particular for legumes, to recruit at undisturbed plots. Most likely, both effects were indirectly mediated by their strong negative effects on aboveground biomass and thereby reducing competition for light. The consistently positive effect of rodent herbivory on the number of recruiting species, irrespective of their exotic or native origin, opposes several studies that provided evidence for biotic resistance by rodents in North American grassland^13,14^. Major players in these grasslands are granivorous rodents which have been frequently shown to selectively consume large-seeded exotic species, reducing establishment success and abundance of these species. However, in our Central European grassland we primarily find herbivorous rodents which do not selectively feed on large-seeded exotic species. From our study, we might suggest that herbivorous rodents rather facilitate the establishment of exotic (and native) species particularly at undisturbed plots likely by reducing completion for light. These findings may have important implications for our understanding of biological invasions and suggest that the fate of exotic species recruitment will depend on which herbivore guild dominates in a grassland system (i.e. facilitative effects of herbivorous rodents vs. suppressive effects of gastropods and granivorous rodents).

### What are the consequences for species diversity, composition and productivity of plant communities experimentally invaded by exotic and native species?

Against expectations, changes in the density and number of recruiting species, as a result of the interactive effect between gastropod herbivory, disturbance and species origin, did not result in changes in species richness, composition and productivity. Probably this is related to the ability of plants to tolerate herbivore damage^47^. However, we found that rodent and gastropod herbivory showed opposing and interactive effects on species richness and community composition, an effect which was not seen during seedling recruitment. The positive effect of rodents on species richness, most likely indirectly mediated via strong negative effects on aboveground biomass, became only visible in the absence of gastropods that were feeding on sub-dominant species (particularly legumes), which benefitted from the release of aboveground competition in the presence of rodent herbivory. Allan and Crawley^23^ found non-additive effects for gastropods and insects: insects were feeding on dominant grasses, thereby increased species richness, but only if gastropods were absent as they suppressed sub-dominant herb or legume species. The opposing effects of gastropods and rodents on diversity may be related to their difference in body size. Compared to smaller sized herbivores, larger herbivores are frequently less selective in food choice, but have stronger effects on the performance or productivity of plants^48,49^. In our experiment, gastropods particularly reduced the numbers and abundance of legume species, known to be highly nutritious. This gastropod x rodent interaction was, however, not evident during early recruitment, where the effects of both herbivore guilds were independent of each other.

Finally, in agreement with our third hypothesis and the study by Maron, *et al*.^27^, addition of exotic species increased productivity to a greater extent than addition of native species, although this difference was only marginally significant. However, in contrast to our third hypothesis and findings by Maron, *et al*.^27^, the effect of exotic vs. native species on productivity was neither amplified by disturbance nor reduced by generalist herbivory. Independent of any alteration in resource availability caused by disturbance, exotic species may thus possess higher aboveground growth rates^50^, which is in line with observational studies finding increased productivity at invaded sites compared to uninvaded ones^1^. Moreover, the positive effect of seed addition on total species richness demonstrates that our grassland community was not saturated with species due to dispersal and seed limitation which frequently occurs in grassland systems^51,52^. As the effect of seed addition on species richness and composition was enhanced by disturbance, we suggest that dispersal limitation in combination with microsite limitation shape species richness and composition in our rather productive grassland community^6^. However, the magnitude of this disturbance x seed addition effect on species richness did not differ between species origins, thus resident native species mediate biotic resistance^9^ but similarly for native and exotic species^27^ but see^53^. A surprising result was that fewer exotic than native species were able to establish themselves in the absence of experimental disturbance, with the consequence that adding seeds of native species resulted in a stronger increase in species richness particularly at undisturbed plots. This result may indicate a stronger niche complementarity between resident species and native colonizers, possibly resulting from a common co-evolution^54^. This also supports the finding by Breitschwerdt, *et al*.^52^, that species which commonly co-occur with the resident vegetation have the better chances to colonize. Finally, our result that exotic species colonized in fewer numbers but produced more biomass is consistent with other experimental results suggesting that mechanisms driving the diversity-productivity relationship differ between native and exotic-dominated communities^37–39,41^.

## Conclusions

Our multi-factorial experiment revealed major insights in how disturbance, herbivory by gastropods and rodents, and plant species origin separately, and interactively, affected seedling recruitment, biodiversity and ecosystem functioning, and thus advances our general understanding of ecological processes driving community assembly and dynamics. Quality and quantity of interactions between herbivores, disturbance and species origin differed, depending on whether seedling recruitment, community species richness, composition and productivity were considered as response. For example, during early establishment selective gastropod herbivory conferred biotic resistance particularly against exotic plant species, even balancing the positive influence of disturbance. No such herbivore x disturbance x species origin interaction could be observed for other properties of the plant community. The primary factor influencing how many exotic and native species colonized was disturbance, suggesting that biotic resistance through native competitors is a major determinant of colonization success of introduced plant species. However, the fewer exotic species which were able to colonize still had a stronger impact on the productivity of the community. Our study demonstrates the need to account for the interactions between multiple processes as drivers of early recruitment of invasive species, as well as to investigate underlying mechanisms of biodiversity-ecosystem functioning in exotic dominated “novel” vs. native communities.

## Material and Methods

In 2011, we set up a multi-factorial split-split plot experiment at a grassland site at the field experimental station of the Helmholtz Centre for Environmental Research – UFZ at Bad Lauchstädt, Central Germany (51°23′29.47″N, 11°52′27.76″E). The moderately productive temperate grassland community was characterized by perennial grasses, such as *Arrhenatherum elatius*, *Calamagrostis epigejos* and *Dactylis glomerata*, herbs such as *Taraxacum officinale* and *Plantago major*, and the legumes *Trifolium repens* and *Trifolium pratense*. Abundant rodents were voles (mostly *Microtus arvalis*); in addition hares (*Lepus europaeus*) occurred occasionally. By far the most common gastropods were slugs from the native genus *Deroceras* and the invasive species *Arion vulgaris*^16^. However, recent molecular analyses question the invasive status of *Arion vulgaris* in Europe^55^. The grassland was mown regularly twice a year, in summer and autumn.

**Figure 6:**
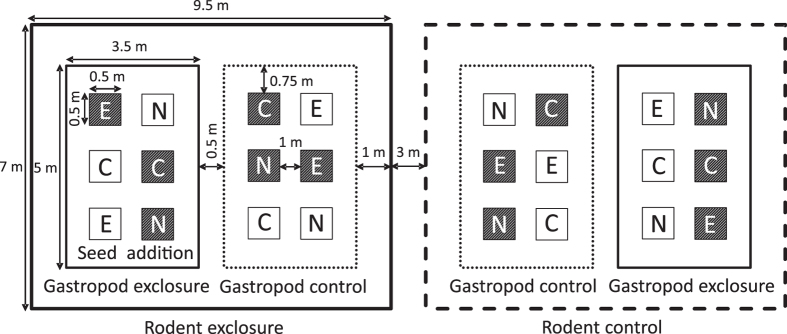
The graph exemplifies the experimental design showing one out of five blocks. Each block includes one rodent exclusion plot (indicated by a bold solid line) and rodent control plot (indicated by a bold dashed line) plot. Within each plot, one gastropod exclusion subplot (indicated by a solid line) and gastropod control subplot (indicated by a dashed line) were established. Within each subplot, sub-subplots (depicted with smaller squares) were established that were either subjected to disturbance (shaded squares) or were left undisturbed (open squares). These sub-subplots were furthermore randomly assigned to receive no seeds (C), a seed mixture of 20 exotic species (E) or 20 native species (N).

The split-split plot experiment was aranged in five blocks and included the experimental factors rodent herbivory, gastropod herbivory, disturbance and seed addition. Each of the five blocks contained a rodent exclosure and a rodent control plot. Within each of these plots one gastropod exclosure and gastropod control was set-up, summing up to each 10 gastropod exclosure and control subplots. Within each subplot we established six 0.5 × 0.5 m experimental sub-subplots. Each of the sub-subplots was randomly assigned to one of the six combinations of disturbance (yes/no) x seed-addition (no seeds, native or exotic seed mixture) (Fig. 6), summing up to a total of 120 sub-subplots.

Rodent exclosures (7 × 9.5 m) were constructed using a fence made of 6 mm mesh wire, buried 0.4 m belowground and extending 0.6 m aboveground, and topped with metal flashing to prevent rodents from climbing into the exclosure. We used bait boxes equipped with rodenticide to eliminate any rodents that might have entered the rodent exclosures. Gastropod exclosures (5 × 3.5 m) were constructed of a metal sheet, buried 0.1 m belowground and extending 0.2 m aboveground. The upper edge of the metal was bent outward and lubricated with lemon-based slug repellent (IRKA® “Schneckenabwehrpaste”, Germany), to prevent gastropods from climbing into the fence. Inside the exclosure we displayed slug baits based on metaldehyde (Spiess-Urania® “Schneckenkorn”) to kill gastropods which entered exclosures. Despite there were no infrastructure controls, we think that the side effects of our exclosures were rather negligible. First, there was a 1.75 m buffer strip between the bigger rodent exclosures and the experimental plots and a 0.75 m buffer strip between the smaller gastropod exclosures and the experimental plots, to keep shade effects as minimal as possible (see Fig. 6). Second, there was no obvious difference between rodent activity inside and outside gastropod exclosures. Moreover, we noticed herbivory by other herbivores, such as deer and aphids, inside and outside our exclosures.

In late October 2011, sub-subplots either received no seeds (control), a native or an exotic seed mixture each consisting out of 20 species. Out of the sown species pool the native species *Daucus carota*, *Dactylis glomerata, Bromus hordeaceus, Vicia tetrasperma* and the exotic species *Solidago canadensis* were already present in the grassland community albeit at low abundance, while all other exotic and native species were absent from that site. Native and exotic species pools were kept as similar as possible with respect to distribution of seed size, life span and functional group identity (Table S1). Seeds of all species were collected in wild-growing populations in Central Germany in summer and autumn 2011 just prior to the start of the experiment. Seeds were cleaned and weighed to determine mean propagule weight per species. To account for the fact that small-seeded species typically produce more seeds than large-seeded species we added 50, 100 or 175 seeds per species depending on their propagule weight (Table S1). Prior to adding seeds, half of the sub-sub-subplots were disturbed by removing the aboveground vegetation and turning the top 0.15 to 0.20 m of soil to reduce competition from resident vegetation.

### Measurements

The identity and number of seedlings of all species was determined on all 0.25 m^2^ sub-subplots in April 2012, including control sub-subplots to account for spontaneous recruitment from the seed bank and colonization from surrounding vegetation. Moreover, we quantified how our treatments subsequently influenced total species richness and composition of the studied grassland community in summer 2012 and 2013. At every census date we recorded the abundance of all species occurring on a certain sub-subplot including control sub-subplots. Central European semi-natural, extensively used grasslands need to be managed to be maintained and conserved, because otherwise succession will take place and bushes will encroach. Imitating the common management practice of such grasslands, we harvested aboveground biomass in July and September, both in 2012 and 2013. Bagged samples were dried at 60 °C for 48 hours and weighed.

### Statistical analysis

#### How do disturbance and the different herbivore guilds, separately and interactively, affect the establishment of exotic vs. native plant species?

To examine how rodent herbivory, gastropod herbivory and disturbance and the affiliation to functional groups affected seedling recruitment of sown species, we calculated the number of exotic and native species producing seedlings on the 0.25 m^2^ sub-subplot minus their values at the respective control sub-subplot. As residuals were already normally distributed, we used the untransformed data of the number of species with seedling recruitment as response variable and applied generalized linear mixed model with Gaussian error distribution. Rodent exclusion, gastropod exclusion, disturbance, species origin were considered fixed factors. Following the split-split-plot design of our experiment, block, block x plot (mainplot error) and block x plot x subplot (subplot error) were included as random effects in the model. The effects of disturbance, seed addition and their interactions were tested against the residual error term.

For those sown exotic and native species with successful recruitment (see above), we calculated the difference between seedling numbers in seed addition sub-subplots and those in control sub-subplots to account for spontaneous colonization from the seed bank or from the surrounding vegetation. We then used the corrected numbers of recruited individuals in relation to the number of added propagules per species as response variable (i.e. proportion of seedling recruitment). Because a binomial model did not converge, data were logit-transformed and linear mixed models were applied^56^. Rodent exclusion, gastropod exclusion, disturbance, species origin and functional group were considered fixed factors, while species nested within functional group and species origin were considered random factors. Moreover, following the split-split-plot design of our experiment, block, block x plot (mainplot error) and block x plot x subplot (subplot error) were included as random effects in the model. The effects of disturbance, seed addition and their interactions were tested against the residual error term. For the two response variables (number of species with seedling recruitment and the proportion of seedling recruitment) only sub-subplots with exotic and native species addition were included in the analysis. Moreover, in case of significant interactions we tested simple main effects (i.e. the effect of one factor at each level of the other factors^57,58^; using the SLICE option in SAS.

#### What are the consequences for species diversity, composition and productivity of plant communities experimentally invaded by exotic and native species?

In contrast to the analysis on seedling recruitment, all sub-subplots including the controls were incorporated in the analysis of total species richness and productivity. Total species richness per sub-subplot was calculated from the number of sown plus resident species. To distinguish how the different functional groups (i.e. grasses, herbs, legumes) responded to our experimental treatments we also calculated species richness including resident species for the functional groups separately. Productivity was assessed as the sum of both aboveground biomass harvests (July and September). For the statistical analysis total species richness and species richness of the three functional groups were square root-transformed while productivity was log-transformed. We used separate repeated-measures linear mixed models to test the effect of our experimental treatments on species richness (i.e. total, grass, herb and legume species richness) as well as aboveground productivity. Here, block, block x plot and block x plot x subplot were considered random effects in the between-subject part of the model, while the two replicates in time (2012, 2013) were considered random in the within subject part of the model. Sub-subplots were tested against residuals of the between subject model. We used orthogonal contrasts to test the *a priori* expectations and decompose significant interactions. Following our third hypothesis, for aboveground biomass and total species richness we tested whether seed addition sub-subplots differed from seed addition control sub-subplots, whether exotic seed addition differed from native seed addition and whether this effect was enhanced by disturbance.

To determine the effects of all experimental treatments on the species composition in 2012 and 2013 we applied different multivariate analysis techniques based on the abundances of recorded species, separately by census year. We calculated permutational multivariate analysis of variance (PERMANOVA). Rodent herbivory, gastropod herbivory, disturbance and species origin were considered fixed effects while block was considered random (“strata”). Differences in vegetation composition in response to treatments were visualized by using nonmetric multidimensional scaling (NMDS) based on Bray-Curtis dissimilarities and k = 4 dimensions. Furthermore, we calculated indicator species analysis (INDVAL) to determine which species were associated with our experimental treatments. Both PERMANOVA and INDVAL analysis were based on 999 permutations. All multivariate analysis were calculated with R 3.0.1 using the vegan^59^ and indicspecies package^60^, while all other analyses were done with SAS 9.2 using the GLIMMIX procedure.

## Electronic supplementary material

Supplementary Information
