# Author Correction: Interactions count: plant origin, herbivory and disturbance jointly explain seedling recruitment and community structure

**DOI:** 10.1038/s41598-018-22518-z

**Published:** 2018-03-08

**Authors:** Lotte Korell, Birgit R. Lang, Isabell Hensen, Harald Auge, Helge Bruelheide

**Affiliations:** 1 Institute of Biology, Martin Luther University Halle-Wittenberg, Am Kirchtor 1, D-06108 Halle, Germany; 2 Department of Community Ecology, Helmholtz Centre for Environmental Research (UFZ), Theodor-Lieser-Straße 4, D-06120 Halle, Germany; 3 German Centre for Integrative Biodiversity Research (iDiv) Halle-Jena-Leipzig, Deutscher Platz 5e, D-4103 Leipzig, Germany; 4 Institute of Special Botany, Philosophenweg 16, D-07743 Jena, Germany

Correction to: *Scientific Reports* 10.1038/s41598-017-08401-3, published online 15 August 2017

This Article contains an error in Figure 5, where the ‘undisturbed’ and ‘disturbed’ bars for the control sample are reversed. The correct Figure 5 appears below as Figure 1.

**Figure 1:**
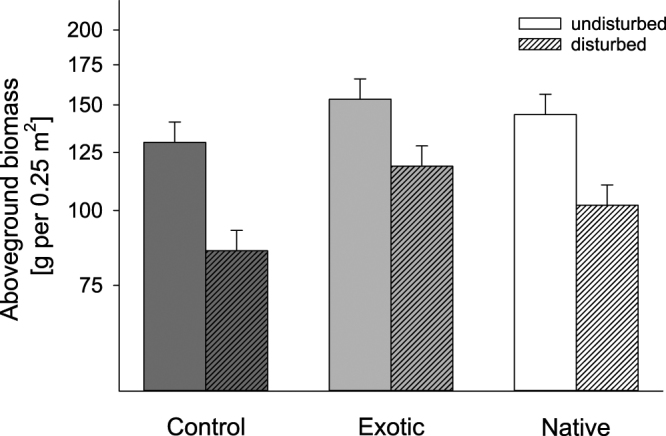
Effect of species origin (control = no seed addition, exotic = exotic seed addition, native = native seed addition) and disturbance (undisturbed, disturbed) on aboveground biomass. Data shown are least square means of repeated-measure linear mixed models ± SE averaged across two years. Note that aboveground biomass is given on a logarithmic scale.

