# Supplementary material for: Interactions count: plant origin, herbivory and disturbance jointly explain seedling recruitment and community structure
Source: Sci Rep. 2017 Aug 15;7:8288. doi: 10.1038/s41598-017-08401-3 (PMC5557803; doi:10.1038/s41598-017-08401-3)
Supplement: Supplementary file 1 — Supplementary Information [file 41598_2017_8401_MOESM1_ESM.pdf]

Interactions count: plant origin, herbivory and disturbance jointly explain seedling recruitment and community structure

Lotte Korell[1,2,3,] Birgit R. Lang [4], Isabell Hensen[1,3], Harald Auge [2,3], Helge Bruelheide [1,3]

[1]Institute of Biology, Martin Luther University Halle-Wittenberg, Am Kirchtor 1, D-06108 Halle, Germany

[2]Department of Community Ecology, Helmholtz Centre for Environmental Research (UFZ), Theodor-Lieser-Straße 4, D-06120 Halle, Germany

[3]German Centre for Integrative Biodiversity Research (iDiv) Halle-Jena-Leipzig, Deutscher Platz 5e, D-4103, Leipzig, Germany

[4] Institute of Special Botany, Philosophenweg 16, D-07743 Jena, Germany

Table S1: Results of a permutational multivariate analysis of variance for the effect of rodent herbivory (rodent control, rodent exclusion), gastropod herbivory (gastropod control, gastropod exclusion), species origin (no seed addition control = C, exotic seed addition = E, native seed addition = N), and disturbance (disturbed = +D, undisturbed = -D) on the species composition within the first and second year of the study. *D.f.* gives the numerator and denominator degrees of freedom. Significant F values are shown in bold: + $P < 0.10$, * $P < 0.05$, ** $P < 0.01$, *** $P < 0.001$.

		**Species composition**	
		1st year	2nd year
**Fixed effects**	*D.f.*	*pseudo-F* ratio	*pseudo-F* ratio
Rodent (ROD)	1, 95	**1.73+**	**3.44*** **
Gastropod (GAS)	1, 95	1.3	**1.75***
Species origin (SO)	2, 95	**9.28*** **	**8.97*** **
Disturbance (DIS)	1, 95	**14.96*** **	**8.12*** **
ROD x GAS	1, 95	**1.56+**	1.31
ROD x SO	2, 95	0.54	0.58
ROD x DIS	1, 95	0.86	0.57
GAS x SO	2, 95	0.89	0.88
GAS x DIS	1, 95	0.97	1.24
SO x DIS	2, 95	**3.95*** **	**3.35*** **
ROD x GAS x SO	2, 95	1.19	0.56
ROD x GAS x DIS	1, 95	1.37	0.53
ROD x SO x DIS	2, 95	0.7	1.05
GAS x SO x DIS	2, 95	0.97	0.8
ROD x GAS x SO x DIS	2, 95	0.53	0.38

Fig. S1

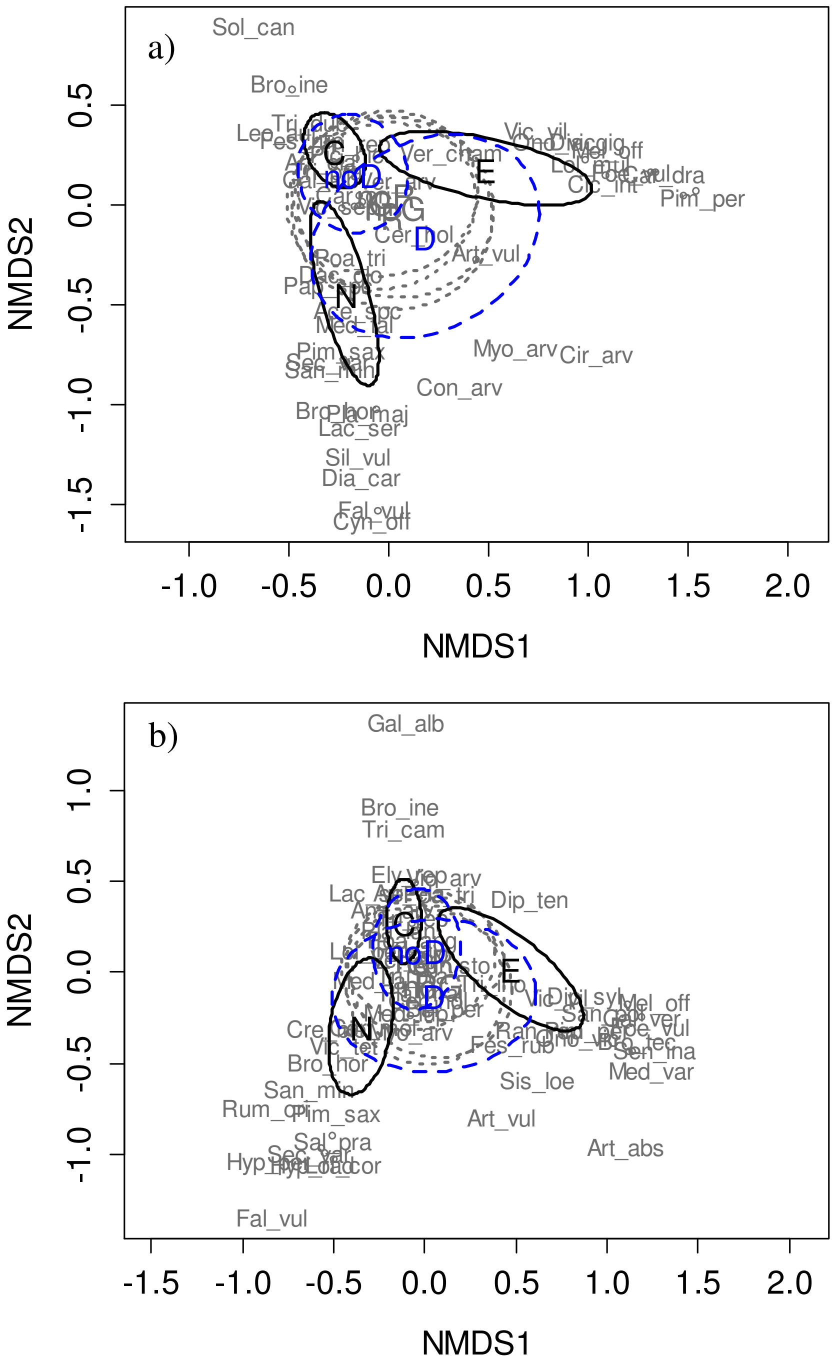

Figure S2: NMDS diagrams showing differences in species composition between rodent herbivory (R) and exclusion (noR), gastropod herbivory (G) and exclusion (noG) indicated by grey color, disturbed (D) and undisturbed (noD) indicated by blue color, and no (C), exotic (E), and native (N) seed addition treatments indicated by black color in the two study years 2012 (a) and 2013 (b). Polygons show the group means and standard errors of the average of site scores. Analyses were based on Bray-Curtis dissimilarity and k=4 dimensions (NMDS stress 2012 = 0.12, NMDS stress 2013 = 0.13).

Table S2: Results of the indicator species analysis (INDVAL) for the association of species top experimental treatments rodent herbivory (rodent control, rodent exclusion), gastropod herbivory (gastropod control, gastropod exclusion), disturbance (undisturbed, disturbed) and species origin (control = no seed addition, exotic = exotic seed addition, native = native seed addition) for the study years 2012 and 2013. Indicator values (indval) of 1 = perfect indication, significance levels: ***p < 0.001, **p < 0.01, *p < 0.05.

	2012			2013		
**Treatment**	**Species**	**Indval**	**Sign.**	**Species**	**Indval**	**Sign.**
**Rodent herbivory**						
control	*Achillea millefolium*	0.45	*	*Lactuca serriola*	0.36	***
exclusion	*Trifolium dubium*	0.56	***	*n.s*	n.s	n.s
**Gastropod herbivory**						
control	n.s	n.s	n.s	*n.s*	n.s	n.s
exclusion	n.s	n.s	n.s	*Trifolium pratense*	0.54	***
**Disturbance**						
undisturbed	*Plantago lanceolata*	0.79	***	*Calamagrostis epigejos*	0.68	***
	Calamagrostis epigejos	0.74	***	*Trifolium repens*	0.58	**
	Trifolium repens	0.66	***	*Trifolium dubium*	0.36	**
	Trifolium dubium	0.50	**			
	Trifolium pratense	0.46	*			
disturbed	*Dianthus gigantheus*	0.56	***	*Dianthus gigantheus*	0.49	**
	Bromus hordeaceus	0.56	***	*Dipsacus sylvestris*	0.48	*
	Bromus tectorum	0.53	***	*Sanguisorba minor*	0.47	*
	Lolium multiflorum	0.51	***	*Lolium multiflorum*	0.46	**
	Cardaria drabra	0.50	***	*Cichorium intybus*	0.45	**
	Dipsacus sylvestris	0.49	***	*Bromus tectorum*	0.44	**
	Elymus repens	0.48	***	*Hypericum perforatum*	0.41	**
	Dianthus carthusianorum	0.45	***	*Dianthus carthusianorum*	0.41	**
	Silene vulgaris	0.43	***	*Silene vulgaris*	0.40	**
	Senecio inaequidens	0.39	***	*Cardaria drabra*	0.40	***
	Medicago x varia	0.37	*	*Tragopogon dubius*	0.36	**
	Cynoglossum officinale	0.36	**	*Elymus repens*	0.33	*
	Diplotaxis tenuifolia	0.36	**			

	**2012**			**2013**		
**Treatment**	**Species**	**Indval**	**Sign.**	**Species**	**Indval**	**Sign.**
disturbed	*Cichorium intybus*	0.35	*			
	Veronica arvensis	0.35	*			
	Lactuca serriola	0.34	*			
	Hypochaeris radicata	0.34	**			
	Foeniculum vulgare	0.32	*			
	Falcaria vulgaris	0.31	*			
	Pimpinella peregrina	0.31	*			
	Sisymbrium loeseli	0.29	*			
	Veronica chamaedrys	0.29	*			
**Seed addition**						
control	n.s	n.s	n.s	*Trifolium dubium*	0.36	*
				Vicia sepium	0.35	*
exotic	*Vicia villosa*	0.89	***	*Dipsacus sylvestris*	0.81	***
	Sanguisorba minor spp. polygama	0.82	***	*Sanguisorba minor spp. polygama*	0.79	***
	Lolium multiflorum	0.70	***	*Dianthus giganteus*	0.68	***
	Dipsacus sylvestris	0.70	***	*Pimpinella peregrina*	0.67	***
	Dianthus giganteus	0.68	***	*Vicia villosa*	0.67	***
	Bromus tectorum	0.67	***	*Lolium multiflorum*	0.66	***
	Onobrychis viciifolia	0.64	***	*Cichorium intybus*	0.63	***
	Cardaria draba	0.61	***	*Bromus tectorum*	0.59	***
	Cichorium intybus	0.54	***	*Onobrychis viciifolia*	0.54	***
	Medicago x varia	0.50	***	*Cardaria drabra*	0.50	***
	Senecio inaequidens	0.47	***	*Senecio inaequidens*	0.44	***
	Diplotaxis tenuifolia	0.44	***	*Medicago x varia*	0.41	***
	Foeniculum vulgare	0.44	***	*Foeniculum vulgare*	0.35	*
	Pimpinella peregrina	0.38	***			
	Melilotus officinalis	0.35	***			

	2012			2013		
**Treatment**	**Species**	**Indval**	**Sign.**	**Species**	**Indval**	**Sign.**
native	*Sanguisorba minor*	0.87	***	*Agrimonia eupatoria*	0.94	***
	Agrimonia eupatoria	0.76	***	*Bromus hordeaceus*	0.89	***
	Bromus hordeaceus	0.75	***	*Vicia tetrasperma*	0.88	***
	Tragopogon dubius	0.68	***	*Sanguisorba minor*	0.80	***
	Dactylis glomerata	0.62	**	*Dactylis glomerata*	0.80	***
	Pimpinella saxifraga	0.57	***	*Pimpinella saxifraga*	0.70	***
	Dianthus carthusianorum	0.54	***	*Cynoglossum officinale*	0.63	***
	Silene vulgaris	0.52	***	*Dianthus carthusianorum*	0.63	***
	Securigera varia	0.50	***	*Hypocheris radicata*	0.57	***
	Vicia tetrasperma	0.47	***	*Securigera varia*	0.57	***
	Lactuca serriola	0.45	***	*Silene vulgaris*	0.50	***
	Cynoglossum officinale	0.44	***	*Tragopogon dubius*	0.44	**
	Hypocheris radicata	0.41	**	*Salvia pratensis*	0.41	**
	Falcaria vulgaris	0.38	*			
	Lotus corniculatus	0.35	**			

Table S3: Native and exotic species added to seed addition subplots (0.25 m^2). Life span is indicated as P = perennial, A = annual, A-B = annual/biennial, A-P = annual/perennial. Affiliation to functional groups is indicated as G = grass, L = legume, NL = non-legume herb

		Life span	Functional group	Propagule mass [mg]	Seed number/ subplot	Seedmass / subplot [g]	Seedling recruitment in April 2012 (mean number of individuals per subplot)
**Exotic species**							
1	*Artemisia absinthium*	P	NL	0.08	175	0.01	0.68
2	*Bromus tectorum*	A	G	6.13	100	0.61	10.5
3	*Bunias orientalis*	P	NL	39.37	50	1.97	0
4	*Cardaria drabra*	P	NL	1.5	100	0.15	7.67
5	*Cichorium intybus*	P	NL	1.41	100	0.14	2.57
6	*Dianthus giganteus*	P	NL	1.23	100	0.12	5.92
7	*Diplotaxis tenuifolia*	P	NL	0.31	175	0.05	3.72
8	*Dipsacus sylvestris*	B	NL	3.24	100	0.32	1.47
9	*Echinops sphaerocephalus*	B	NL	13.98	50	0.7	0
10	*Foeniculum vulgare*	B-P	NL	5.58	100	0.56	0.45
11	*Lolium multiflorum*	B-P	G	7.6	100	0.76	8.67
12	*Medicago x varia*	P	L	1.87	100	0.19	0
13	*Melilotus officinalis*	A-B	L	2.54	100	0.25	0
14	*Onobrychis viciifolia*	P	L	20.1	50	1	3.12
15	*Pimpinella peregrina*	P	NL	3.01	175	0.53	0.35
16	*Sanguisorba minor* ssp. *polygama*	P	NL	13.34	50	0.67	9.62
17	*Senecio inaequidens*	P	NL	0.28	175	0.05	1.22
18	*Sisymbrium loeseli*	A	NL	0.09	175	0.02	0
19	*Solidago canadensis*	P	NL	0.04	175	0.01	0
20	*Vicia villosa*	A	L	39.11	25	0.98	2.95
**Native species**							
1	*Agrimonia eupatoria*	P	NL	26.2	50	1.31	0.22
2	*Bromus hordeaceus*	A	G	2.35	100	0.24	9.07
3	*Cynoglossum officinale*	B	NL	28.01	50	1.4	0.82
4	*Dactylis glomerata*	P	G	0.64	175	0.11	3.02
5	*Daucus carota*	P	NL	0.87	175	0.15	3.27
6	*Dianthus carthusianorum*	P	NL	0.45	175	0.08	4.4
7	*Falcaria vulgaris*	P	NL	1.14	100	0.11	1.75
8	*Hypericum perforatum*	P	NL	0.11	175	0.02	0
9	*Hypochaeris radicata*	P	NL	0.62	175	0.11	1.58
10	*Lactuca serriola*	A	NL	0.46	175	0.08	0.29
11	*Lotus corniculatus*	P	L	0.96	175	0.17	0.54
12	*Medicago falcata*	P	L	0.92	175	0.16	0.62
13	*Pimpinella saxifraga*	B-P	NL	0.62	175	0.11	7.22
14	*Rumex crispus*	P	NL	2.72	100	0.27	0.32
15	*Salvia pratensis*	B-P	NL	1.26	100	0.13	0.25
16	*Sanguisorba minor*	P	NL	7.91	100	0.79	7.3
17	*Securigera varia*	P	L	6.05	100	0.6	0.37
18	*Silene vulgaris*	P	NL	0.62	175	0.11	3.45
19	*Tragopogon dubius*	B	NL	6.52	100	0.65	3.02
20	*Vicia tetrasperma*	A	L	3.4	100	0.34	0.3

Table S3: List of species found in the study with acronyms considered in the NMDS.

No.	Species	Acronym
1	*Achillea millefolium*	Ach _mil
2	*Agrimonia eupatoria*	Agr _eup
3	*Agrostis stolonifera*	Agr _sto
4	*Anagallis arvensis + foemina*	Ana _arv
5	*Arrhenatherum elatius*	Arr _ela
6	*Artemisia absinthium*	Art _abs
7	*Artemisia vulgaris*	Art _vul
8	*Bellis perennis*	Bel _per
9	*Bromus hordeaceus*	Bro _hor
10	*Bromus inermis*	Bro _ine
11	*Bromus tectorum*	Bro _tec
12	*Bunias orientalis*	Bun _ori
13	*Calamagrostis epigejos*	Cal _epi
14	*Cardaria draba*	Car _aca
15	*Carduus acanthoides*	Car _cri
16	*Carduus crispus*	Car _dra
17	*Cerastium holosteoides*	Cer _hol
18	*Cichorium intybus*	Cic _int
19	*Cirsium arvense*	Cir _arv
20	*Convolvulus arvense*	Con _arv
21	*Crepis biennis*	Cre _bie
22	*Cynoglossum officinale*	Cyn _off
23	*Dactylis glomerata*	Dac _glo
24	*Daucus carota*	Dau _car
25	*Dianthus carthusianorum*	Dia _car
26	*Dianthus giganteus*	Dia _gig
27	*Diplotaxis tenuifolia*	Dip _syl
28	*Dipsacus sylvestris*	Dip _ten
29	*Echinops sphaerocephalus*	Ech _sph
30	*Elymus repens*	Ely _rep
31	*Epilobium tetragonum*	Epi _tet
32	*Falcaria vulgaris*	Fal _vul
33	*Festuca rubra*	Fes _rub
34	*Foeniculum vulgare*	Foe _vul
35	*Galium album*	Gal _alb
36	*Galium verum*	Gal _ver
37	*Geranium molle*	Ger _mol
38	*Hypericum perforatum*	Hyp _per
39	*Hypocheris radicata*	Hyp _rad
40	*Lactuca serriola*	Lac _ser
41	*Leontodon autumnalis*	Leo _aut
42	*Lolium multiflorum*	Lol _mul
43	*Lolium perenne*	Lol _per
44	*Lotus corniculatus*	Lot _cor

45	*Medicago falcata*	Med _fal
46	*Medicago lupolina*	Med _lup
47	*Medicago x varia*	Med _var
48	*Melilotus officinalis*	Mel _off
49	*Myosotis arvensis*	Myo _arv
50	*Onobrychis viciifolia*	Ono _vic
51	*Papaver spec.*	Pap _spc.
52	*Picris hieracioides*	Pic _hie
53	*Pimpinella peregrina*	Pim _per
54	*Pimpinella saxifraga*	Pim _sax
55	*Plantago lanceolata*	Pla _lan
56	*Plantago major*	Pla _maj
57	*Poa angustifolia*	Poa _ang
58	*Poa pratensis*	Poa _pra
59	*Poa trivialis*	Poa _tri
60	*Polygonum aviculare*	Pol _avi
61	*Ranunculus repens*	Ran _rep
62	*Rumex crispus*	Rum _cri
63	*Salvia pratensis*	Sal _pra
64	*Sambucus nigra*	Sam _nig
65	*Sanguisorba minor*	San _min
66	*Sanguisorba minor subsp. polygama*	San _pol
67	*Securigera varia*	Sec _var
68	*Senecio inaequidens*	Sen _ina
69	*Silene vulgaris*	Sil _vul
70	*Sisymbrium loeseli*	Sis _loe
71	*Taraxacum officinale*	Tar _off
72	*Tragopogon dubius*	Tra _dub
73	*Trifolium campestre*	Tri _cam
74	*Trifolium dubium*	Tri _dub
75	*Trifolium pratense*	Tri _ino
76	*Trifolium repens*	Tri _pra
77	*Tripleurospermum inodrum*	Tri _rep
78	*Veronica arvensis*	Ver _arv
79	*Vicia sepium*	Vic _sep
80	*Vicia tetrasperma*	Vic _tet
81	*Vicia villosa*	Vic _vil
82	*Viola arvensis*	Vio _arv
